# A Review on Recent Progress in Preparation of Medium-Temperature Solar-Thermal Nanofluids with Stable Dispersion

**DOI:** 10.3390/nano13081399

**Published:** 2023-04-18

**Authors:** Ting Hu, Jingyi Zhang, Ji Xia, Xiaoxiang Li, Peng Tao, Tao Deng

**Affiliations:** State Key Laboratory of Metal Matrix Composites, School of Materials Science and Engineering, Shanghai Jiao Tong University, 800 Dong Chuan Road, Shanghai 200240, China

**Keywords:** solar-thermal nanofluids, dispersion stability, medium-temperature nanofluid, solar collector, stabilization mechanism

## Abstract

Direct absorption of sunlight and conversion into heat by uniformly dispersed photothermal nanofluids has emerged as a facile way to efficiently harness abundant renewable solar-thermal energy for a variety of heating-related applications. As the key component of the direct absorption solar collectors, solar-thermal nanofluids, however, generally suffer from poor dispersion and tend to aggregate, and the aggregation and precipitation tendency becomes even stronger at elevated temperatures. In this review, we overview recent research efforts and progresses in preparing solar-thermal nanofluids that can be stably and homogeneously dispersed under medium temperatures. We provide detailed description on the dispersion challenges and the governing dispersion mechanisms, and introduce representative dispersion strategies that are applicable to ethylene glycol, oil, ionic liquid, and molten salt-based medium-temperature solar-thermal nanofluids. The applicability and advantages of four categories of stabilization strategies including hydrogen bonding, electrostatic stabilization, steric stabilization, and self-dispersion stabilization in improving the dispersion stability of different type of thermal storage fluids are discussed. Among them, recently emerged self-dispersible nanofluids hold the potential for practical medium-temperature direct absorption solar-thermal energy harvesting. In the end, the exciting research opportunities, on-going research need and possible future research directions are also discussed. It is anticipated that the overview of recent progress in improving dispersion stability of medium-temperature solar-thermal nanofluids can not only stimulate exploration of direct absorption solar-thermal energy harvesting applications, but also provide a promising means to solve the fundamental limiting issue for general nanofluid technologies.

## 1. Introduction

Sustainable development and modernization of human society calls for exploration of renewable energy technologies [1,2,3]. Among various renewable energy sources, solar energy has attracted significant research attention owing to its giant capacity, cleanness, ubiquitous availability, and diverse conversion [4]. Solar-thermal conversion is a facile and efficient way to harness the abundant solar irradiation to generate heat [5], which can be directly used for heating of buildings and water [6,7], driving industrial processes [8,9], or further converted into other forms of energy such as electricity [10] and chemical fuels [11,12,13]. In particular, the large-scale low-cost storage capability of solar-thermal energy, which can overcome the intermittence issue of renewable energy, would endow solar-thermal technologies with unique competitiveness [14,15]. Great efforts had been made to harvest and store the abundant solar-thermal energy as sensible heat, latent heat, or thermochemical energy to adjust the mismatch between energy supply and consumption need [16,17]. Among them, sensible heat storage within flowable liquids has evolved to be the most mature way to harvest, store, and transport solar-thermal energy.

**Figure 1:**
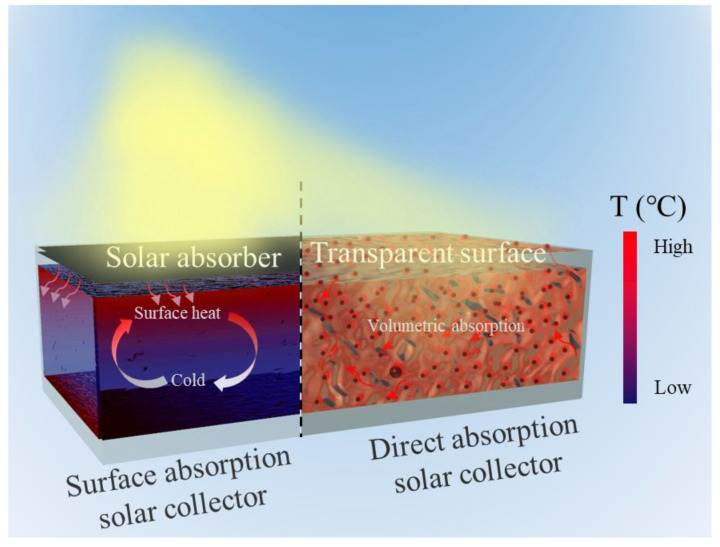
Schematic showing converting concentrated sunlight into medium-temperature heat by surface absorption-based solar collectors and direct absorption-based solar collectors.

Solar collectors, which absorb incident solar irradiation and convert it into storable heat, is one of the key components in solar-thermal systems [18]. As schemed in Figure 1, conventional surface absorption-based solar collectors utilize blackened surfaces or solar selective coatings to efficiently absorb broadband sunlight and generate heat, which is carried away by the flowing heat transfer fluids (HTFs) [19]. However, the transportation of converted heat is often limited by the low thermal conductivity of the HTFs, which causes overheating of the surface coating and sacrifices energy harvesting efficiency [19,20]. In recent years, direct absorption-based solar collectors (DASCs) [21], which make use of homogeneously dispersed photothermal particles to volumetrically harvest solar-thermal energy within the nanofluids, have been pursued as a superior way to harness solar-thermal energy [22,23]. The direct volumetric conversion and storage of solar-thermal energy as sensible heat in the thermal storage fluids avoid the slow heat transfer from the outer surfaces to the inner storage media and homogenize the temperature distribution within the storage media [24]. Such design thus can avoid the potential overheating of the outer surface, and minimize the radiation and convection heat loss from the solar collector, which in turn enables achieving high energy conversion efficiency at medium-to-high temperatures [25]. Such nanofluid-based DASCs not only improve the sustainability of solar-thermal systems [26], but also can be coupled with photovoltaic and solar steam generation systems for multifunctional applications [27].

The performances of DASCs are largely affected by the solar-thermal nanofluids including their dispersion behavior and key thermophysical properties such as solar absorptance, heat capacity, viscosity, and thermal conductivity [28]. In the past, intensive research efforts were directed to improving these thermophysical properties through synthesizing a variety of nanofluids loaded with plasmonic metallic particles and other type of solar absorbers [29], tailoring the loading, morphology, and size of the nanoscale solar absorbers [30], and incorporating multifunctional hybrid nanoparticles [31,32]. So far, however, these prepared solar-thermal nanofluids usually suffer from poor dispersion stability. Once forming agglomeration, not only the nanofluids lose their enhancement of thermophysical properties and volumetric solar-thermal harvesting advantages but also would cause clogging of the DASCs. While previous research efforts were concentrated on low-temperature water-based nanofluids [33,34], in recent years substantial progresses have been made in preparing stably dispersed medium-temperature solar-thermal nanofluids with an application temperature range of 100–400 °C [35,36]. Although elevated service temperatures pose even grand challenges to achieving stable dispersion, these medium-temperature nanofluids hold the promise to provide high-quality renewable heating and expand the application scope of DASCs.

Despite dispersion stability having been viewed as one of the key factors that limit the development of nanofluid technologies and their practical applications, the previous literature was mostly focused on low-temperature aqueous dispersion systems. In recent years, along with other research groups, we have explored effective strategies to improve dispersion stability and understand dispersion behavior of solar-thermal nanofluids at medium temperatures. In this review, we overview state-of-the-art developments of medium-temperature solar-thermal nanofluids and specifically focus on dispersing strategies of these nanofluids. We briefly introduce the desired features of both the thermal storage fluids and solar absorbers for preparing medium-temperature solar-thermal nanofluids. We discuss the specific challenges for dispersing nanoscale solar absorbers within the nanofluids at elevated temperatures. After identifying these challenges, we describe the dispersion strategies that had been explored to improve the dispersion stability and introduce the general working principles of these strategies. We provide comprehensive overview of the dispersion approaches by introducing representative examples that apply these dispersion strategies within ethylene glycol, oil, ionic fluid, and molten salt systems. Finally, we point out the application opportunities, existing problems, and future research directions in the development and application of medium-temperature solar-thermal nanofluids.

## 2. Medium-Temperature Solar-Thermal Nanofluids

**Figure 2:**
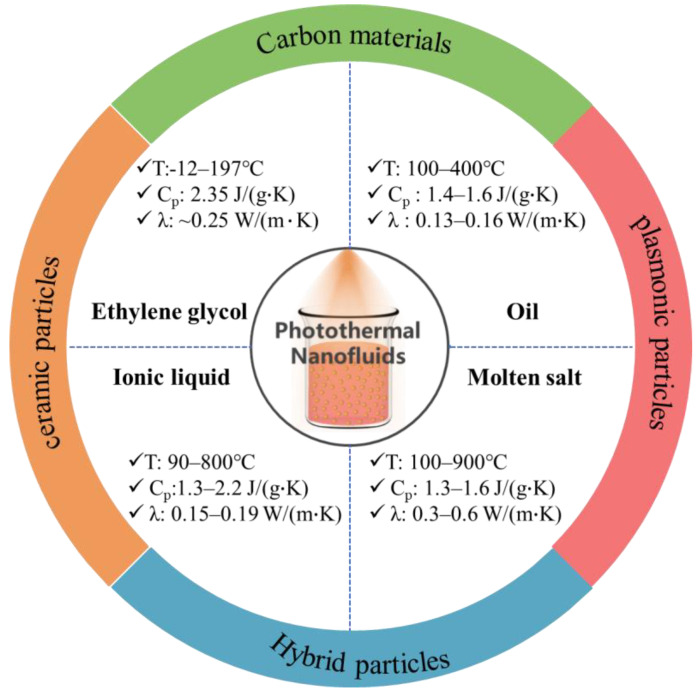
Representative thermal storage fluids and solar absorbers for preparing medium-temrature solar-thermal nanofluids.

The desired nanofluids for direct absorption-based solar-thermal harvesting at medium temperatures should simultaneously possess the following characteristics [37]. Firstly, the nanofluids should have high solar absorptance such that they can efficiently absorb broad-spectrum sunlight and convert it into heat. Secondly, a large heat capacity is desired in order to maximize the solar-thermal energy storage density of the DASCs. Thirdly, the nanofluids should possess a high thermal conductivity to effectively exchange and release the harvested thermal energy to the application terminals. Fourthly, the nanofluids should have a low viscosity similar as that of the thermal storage fluids in order to keep the desired flowability under low mechanical pumping power. In addition, the nanofluids should have good physical and chemical stability under elevated temperatures and concentrated solar illumination conditions. Most importantly, the nanofluids should maintain their stable uniform dispersion under concentrated solar irradiation. These in turn pose strict requirements on the selection of thermal storage fluids and solar absorbers (Figure 2).

Currently, the investigated thermal storage fluids fall into four categories: ethylene glycol, oils (silicone oil, mineral oil), ionic liquids, and molten salts. As a polar working fluid, ethylene glycol can work within a temperature range of −12–197 °C and has a high specific heat capacity of 2.35 J/(g·K) due to its strong molecular hydrogen bonding [38,39]. Silicone oil and other nonpolar synthetic oils have a specific heat capacity of 1.4–1.6 J/(g·K) and can withstand a temperature up to 400 °C [40,41,42]. Owing to the formation of strong ionic bonds between organic cations and anions, the ionic liquids can stably operate under a high temperature up to 800 °C and possess a specific heat capacity of 1.3–2.2 J/(g·K) [43,44]. Although they have demonstrated improved thermal stability, these synthesized ionic liquids are generally expensive at laboratory scales. At industrial scale, the cost of ionic liquids produced has decreased to less than 20 US$/kg due to recent developments [45], which make them attractive for heat transfer and thermal storage. Molten salts with a specific heat capacity in the range of 1.3–1.6 J/(g·K) are the fluids for solar-thermal energy harvesting at even higher temperatures up to 900 °C [46]. To date, synthetic oils (silicone oil, Dowthermal oil) and molten salts (solar salts, Hitec salts) are the main commercial fluids used for harvesting medium-temperature and high-temperature heat in solar-thermal power plants. By comparison, molten salts have cost advantages, but the strong corrosivity and the high freezing point are the main identified issues [47,48]. Corrosion-resistant containers and pipelines made of Ni-based alloys or stainless steels are used in solar-thermal plants [49]. To avoid salt freezing at night, auxiliary heating systems are often installed, and new eutectic salts have been continuously investigated [50,51].

A common feature of commercial thermal storage fluids is that they are transparent, most often colorless, which implies that they have very low solar absorptance. Various solar absorbers had been incorporated into the thermal storage fluids to enable direct absorption-based solar-thermal harvesting. Besides high absorptance of broadband sunlight, one key requirement is that the added solar absorbers can physically and chemically withstand the high service temperature and concentrated solar irradiation. So far, several types of absorbers including carbon nanomaterials [52], plasmonic particles [53], ceramic particles [31], and hybrid particles [54] had been explored as the promising candidates. Among them, carbon absorbers, such as graphite particle [55], reduced graphene oxide (RGO) [56], graphene [57], and carbon nanotube (CNT) [58], are the most popular choice because they are naturally black, stable against solar irradiation, and can be manufactured at large scale with abundant low-cost carbon raw materials such as graphite. In recent years, the plasmonic resonance effect of metallic nanoparticles (Au, Ag) has drawn intensive research attention for efficient photothermal conversion applications [59]. The absorption peak can be tuned through tailoring the size and the morphology of the plasmonic absorbers [60,61], which offers the opportunity to convert broad-spectrum sunlight into intensive plasmonic heat. Ceramic semiconductor particles are another category of solar absorbers, which make use of their small bandgap to absorb incident solar photons and convert them into heat. Most frequently, composition design, defect engineering and particle size are used as the means to tailor the bandgap and thereby the solar-thermal conversion performances of these ceramic solar absorber particles [62]. In comparison with metallic plasmonic particles, ceramic particles such as TiN have exhibited significantly enhanced thermal stability due to their stronger ionic bonding [63]. In addition to single-component absorbers, hybrid particles can further improve solar-thermal conversion efficiency and other thermophysical properties or combine individual functionality from many components [64,65].

## 3. Dispersion Challenge and Stabilization Strategy

### 3.1. Dispersion Challenge at Elevated Temperatures

The dispersion behavior of nanofluids is governed by the particle-particle, and particle-fluid interactions [66]. Most frequently, the added fillers do not have attractive interaction with the thermal storage fluids, i.e., the fillers like each other and prefer to stay closely together to minimize the exposed surface areas and lower the surface energy for the whole system. For the fillers that have large exposed interacting surfaces and strong interparticle attraction such as graphene, it becomes challenging to disperse them homogeneously and stably within the fluids.

If there is no attractive interaction between the fillers and the thermal storage fluid, the ubiquitous attractive van der Waals force would drive the aggregation of the added fillers. The van der Waals interaction force (*F*_vdW_) between two same neighboring spherical particles with a radius of *r* could be described by [67,68]:

(1) $$F_{\mathrm{vdW}}=-\frac{{\left(\sqrt{A_{s}}-\sqrt{A_{l}}\right)}^{2}r}{12L^{2}}$$

where *L* is the distance between two approaching particles, *A*_s_ and *A*_l_ are the Hamaker constants of the dispersed solid particle and the thermal storage fluid, respectively. *A*_s_ is proportional to the square of the atomic density of the particle. As a type of short-range interaction, the van der Waals forces are effective only when the neighboring particles are closely contacting with each other. The high application temperature intensifies the random Brownian motion of the solar absorbers and significantly increases their colliding probability, thereby exacerbating the aggregation tendency. When the particles are approaching each other, their motion is restricted by the friction force between the particle and the thermal storage fluid (*F*_r_). At elevated temperatures, the restriction force decreases as the viscosity of fluid drops, which also intensifies the tendency for neighboring particles to form agglomerates.

For a single particle dispersed within the thermal storage fluid, it is simultaneously subject to the gravity force (*F*_g_), the buoyance force (*F*_b_), and the viscous friction force (*F*_v_). Due to the density difference between the filler and the thermal storage fluid, the heavier inorganic or metallic particle tends to gradually precipitate out of the fluids. The net gravity force (∆F) can be described by [69]:

(2) $$∆F=\frac{4\pi }{3}r^{3}\left(\rho_{f}-\rho_{l}\right)g$$

where $\rho_{s}$ and $\rho_{l}$ are the density of the solid filler particle and the thermal storage fluid, and *g* is the acceleration of gravity (9.8 m/s^2^). According to the Stokes law, the *F*_v_ can be calculated by [70]:

(3) $$F_{v}=6\pi r\eta \nu$$

where η is the viscosity of the nanofluids, and ν is the sedimentation velocity. Based on force balance, the gravitational sedimentation velocity of a colloidal spherical particle (*v*_s_) can be estimated by [71]:

(4) $$\nu_{s}=\frac{2r^{2}\left(\rho_{s}-\rho_{l}\right)g}{9\eta }$$

A smaller particle size, a matching density and a high viscosity are favorable for reducing the sedimentation velocity. When the fluids are working under elevated temperatures, the viscosity dramatically drops, which in turn leads to rapid rise in the sedimentation velocity.

In the meanwhile, the particle is continuously bombed by the thermal storage fluid molecules undergoing random motion. The average Brownian motion velocity (*v*_B_) of the spherical particle within a certain period (*t*) can be estimated by [72,73]:

(5) $$v_{B}=\sqrt{\frac{k_{B}T}{6\pi r\eta t}}$$

where *k*_B_ is the Boltzmann’s constant, *T* is the temperature of the nanofluids in the unit of Kelvin. The particles with a small size are expected to have a high Brownian motion velocity. Increasing temperature, on one hand, can decrease the viscosity of the nanofluids and accelerate the Brownian motion, which is favorable for dispersing single particles. In the meanwhile, the interparticle collision is also intensified at elevated temperatures. When the bare particles are contacting with each other, it had been previously reported that plasmonic solar absorbers such as gold nanoparticles tend to undergo coalescence at elevated temperatures and form large-sized aggregates [74,75]. Long-term heating at elevated temperatures could even cause Ostwald ripening and phase change in the nanoscale solar absorbers within the fluids [76]. For chemically stable carbon-based solar absorbers, it was also found that the nanofluids gradually lost their uniform dispersion with increasing application temperatures. In this case, increasing temperature leads to reduced viscosity of the thermal storage fluids, which cannot provide sufficient restriction forces to prevent gravitational sedimentation of the particles. For the surface-modified particles, it was found that the dynamic adsorption-desorption of surface ligands was also intensified at high temperatures [77]. After losing the protection of the surface ligands, the added particles tend to form agglomerates. The elevated temperature increases the reactivity of surface atoms of the nanofillers, the desorption kinetics of surface-capping agents, and gravitational sedimentation tendency due to reduced viscosity of the thermal storage fluids, which together add more challenges in achieving stable dispersion of medium-temperature solar-thermal nanofluids.

### 3.2. Stabilization Strategy

**Figure 3:**
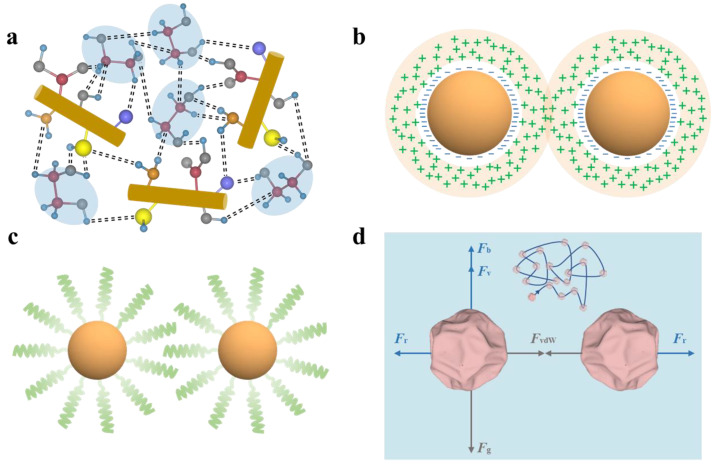
Schematics showing different types of stabilization strategies for preparing medium-temperature solar-thermal nanofluids: (**a**) hydrogen bonding stabilization, (**b**) electrostatic stabilization, (**c**) steric stabilization, (**d**) self-dispersion stabilization.

The key to achieving stable dispersion of medium-temperature solar-thermal nanofluids lies in simultaneously suppressing the van der Waals interparticle attraction and gravitational sedimentation, which can be realized through judiciously selecting the solar absorbers with proper size, density, morphology and tailoring their surface chemistry. As schemed by Figure 3, the stabilization strategies that are applicable to medium-temperature solar-thermal nanofluids can be classified into four categories: hydrogen bonding stabilization, electrostatic stabilization, steric stabilization, and self-dispersion stabilization.

#### 3.2.1. Hydrogen Bonding Stabilization

In polar fluid systems such as ethylene glycol [78], propylene glycol [79] that have hydroxyl groups, they can form hydrogen bonds with solar absorbers that have abundant oxygen-containing surface functional groups. These hydrogen bonds provide the favorable filler-fluid interaction for the solar absorbers to overcome the van der Waals interparticle attraction. Under such circumstance, homogeneous dispersion can be achieved because the solar absorbers and the thermal storage fluid molecules prefer to mix with each other (Figure 3a). While absorbers such as RGO and MXene are naturally rich in oxygen-containing surface functional groups, the surfaces of graphene, CNTs and other solar absorbers can be modified through various kinds of surface modification techniques such as plasma treatment, washing with strong acids or grafting of polar ligands/chains. The number density of these surface functional groups and their bonding strength with the thermal storage fluid molecules affect the dispersion state and dispersion stability of the nanofluids. The key requirements for such stabilization strategy are that the surfaces of solar absorbers should have polar functional groups to form the hydrogen bonding with the thermal storage fluids, and the particle-fluid bonding can withstand the application temperature. It is a facile way to improve the dispersion stability of solar absorbers within polar thermal storage fluids such as ethylene glycol, but it is difficult to apply such stabilization strategy in other nonpolar media such as silicone oil.

#### 3.2.2. Electrostatic Stabilization

By introducing charges with the same polarity onto the surfaces of the solar absorbers through adsorption of ions or charged species, electrostatic repulsion provides the force to counterbalance the interparticle van der Waals attraction. Within the nanofluids, the charged particles are surrounded by counter-ions forming an electrical double layer, which consists of an inner tightly bound Stern layer and an outer diffusive Gouy layer [80]. When the charged particles are approaching, the electrical double layer overlaps and generates the mutual repulsion force (Figure 3b). The thickness of the electrical double layer can be characterized by the Debye length ($L_{D}$), which is expressed as [81,82,83]:

(6) $$L_{D}=\sqrt{\frac{\varepsilon_{0}\varepsilon_{r}k_{B}T}{8\pi ce^{2}z^{2}}}$$

where *c* and *z* are the concentration and the valence of the counter ions, $\varepsilon_{0}$ and $\varepsilon_{r}$ are the permittivity of vacuum and the dielectric constant of the dispersion fluid, and *e* is the unit of charge (1.6 × 10^−19^ C). A low concentration of counter-ions is favorable for forming a thick electrical double layer, which is desired for screening the interparticle van der Waals attraction and stabilizing the dispersion. The electrostatic stabilization is normally effective in polar fluid systems as the readily polarizable fluids have a high dielectric constant, which is favorable for increasing the thickness of the electrical double layer. Experimentally, zeta potential, which is defined as the electrical potential difference between the dispersion fluid and the bounded charge layer onto the dispersed particles, has been widely adopted as a key parameter to evaluate the dispersion stability [84]. Typically, the absolute value of the zeta potential needs to be above a threshold value in order to achieve stable dispersion [85]. Electrostatic stabilization is a popular strategy for improving dispersion of solar absorbers within polar thermal storage fluids, but the dispersion stability can be affected by the addition of other electrolytes or other fillers.

#### 3.2.3. Steric Stabilization

Steric hindrance stabilization is another popular approach to stabilize the dispersion of colloidal particles [86,87]. It relies on surface modification of the particles with organic/inorganic ligands or polymer chains to prevent direct particle contact thereby weakening the van der Waals interparticle attraction (Figure 3c). A variety of surface modification techniques such as ligand exchange and polymer chain grafting have been developed to create the steric protection layer on the surfaces of various solar absorbers. These surface modification agents can be chemically bound or physically adsorbed onto the surface of solar absorbers. The general requirement of the surface modification agents is that they need to be compatible with the thermal storage fluids. One straightforward approach is that the surface modification agents are chemically same as the thermal storage fluids. When the modified particles are dispersed within the fluids, these decorated compatible ligands or polymer chains can be swollen by the thermal storage fluid molecules. The number density, the length distribution of the surface modification agents as well as their binding strength with solar absorbers are the key parameters affecting their dispersion stability. In comparison with hydrogen bonding stabilization and electrostatic stabilization, steric hindrance stabilization can be applied to both polar and nonpolar fluid systems, but it typically involves multi-step complex surface modification processes. When dispersing solar absorbers within polar thermal storage fluids such as ionic fluids and molten salts, steric stabilization and electrostatic stabilization strategies are often combined.

#### 3.2.4. Self-Dispersion Stabilization

In recent years, self-dispersible nanofluids have gained intensive research attentions because the solar absorbers are designed and prepared in such a way that they can be uniformly dispersed within the thermal storage fluids without resorting to complicated post-synthesis surface modification procedures that are usually involved in other stabilization approaches [88]. As schemed by Figure 3d, this strategy makes use of the rough surface of the solar absorber to prevent large-area direction contact between neighboring particles and thus suppress the van der Waals interparticle attraction. Furthermore, the size distribution and the density of the solar absorbers are strictly controlled within a narrow range so that the Brownian motion can counterbalance the gravitational sedimentation. As a general approach, such strategy can be applied to realize homogeneous dispersion of different solar absorbers within both polar and nonpolar thermal storage fluids. The is favorite for promising scalable manufacturing of the solar-thermal nanofluids. To achieve stable dispersion at elevated temperatures, the key challenge of this dispersion strategy lies in controlled synthesis of solar absorbers that simultaneously possess appropriate surface roughness, a sufficiently small particle size, a narrow particle size distribution and a suitable density matching that of the thermal storage fluid. Evaporation-induced crumpling processes, surface etching techniques and many other approaches have been developed to prepare such self-dispersible solar absorbers and tailor their size, surface morphology and density.

## 4. Stably Dispersed Medium-Temperature Solar-Thermal Nanofluids

Depending on the nature of specific thermal storage fluids (ethylene glycol, oil, ionic fluid, and molten salt), various types of solar absorbers have been incorporated to prepare homogeneously dispersed medium-temperature solar-thermal nanofluids by utilizing one of the dispersion strategies or combined ones. Herein, we provide representative research examples showing how these strategies are implemented to improve the dispersion stability of the medium-temperature solar-thermal nanofluids.

### 4.1. Ethylene Glycol-Based Nanofluids

**Figure 4:**
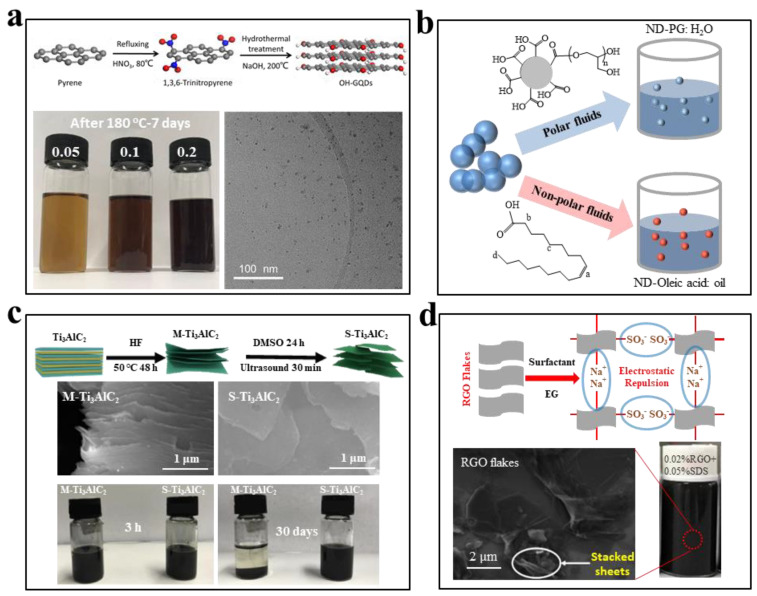
(**a**) The hydrogen bonding between the hydroxyl groups on the surface of GQDs and the ethylene glycol molecules enables homogenous dispersion of nanofluids with different concentrations (0.05 mg/mL, 0.1 mg/mL, 0.2 mg/mL) after heating at 180 °C for 7 days. ((**a**) reprinted/adapted with permission from Ref. [91]. 2019, Royal Society of Chemistry). (**b**) Covalent surface modification of deaggregated nanodiamond particles via formation of poly(glycidol) polymer brush chains to achieve stable dispersion within ethylene glycol. ((**b**) reprinted/adapted with permission from Ref. [92]. 2013, the American Chemical Society). (**c**) Electrostatically stabilized single-layer Ti_3_C_2_T_x_ MXene homogeneously dispersed within ethylene glycol at room temperature for 30 days. ((**c**) reprinted/adapted with permission from Ref. [93]. 2021, Elsevier). (**d**) Uniform dispersion of RGO within ethylene glycol with the assistance of electrostatic stabilization by surfactants. ((**d**) reprinted/adapted with permission from Ref. [94]. 2020, Elsevier).

The hydrogen bonding between the ethylene glycol molecules and the surface functional groups on the surface of solar absorbers provides a facile way to prepare homogeneously dispersed solar-thermal nanofluids. Wang et al. [89] reported that while graphite nanoparticles were not dispersible within ethylene glycol, after acid treatment the graphite oxide nanoparticles (GONs) could retain their stable dispersion for 1 day. They further demonstrated that decorating GONs onto the surfaces of GO sheets could prolong the duration of stable dispersion to 4 weeks, but the dispersion stability of these nanofluids were tested at room temperature. At elevated temperatures, Shu et al. [90] found that while conventional water-wetted multi-layer GO sheets lost their dispersion stability within EG fluids, the ethanol-wetted single-layer GO sheets have demonstrated improved dispersion stability due to the richer oxygen-containing surface functional groups and the smaller mass of dispersed sheets. Through a one-step heating reduction, GO sheets were converted into solar-absorbing RGO, and these RGO sheets could maintain their uniform dispersion after continuous heating at 120 °C for at least 12 h. In a recent work, Lin et al. [91] synthesized hydroxyl-functionalized graphene quantum dots (OH-GQD) with a narrowly distributed size smaller than 10 nm by using a bottom-up method (Figure 4a). Compared with the large-sized GO sheets, the ultrasmall OH-GQDs simultaneously reduced the van der Waals interparticle attraction and mitigated gravitational sedimentation. The resultant EG nanofluids loaded with different concentrations of OH-GQDs could retain their stable dispersion after heating at 180 °C for 7 days and enabled direct harvesting of solar-thermal energy under concentrated solar illumination.

Surface modification of the solar absorbers with organic ligands or polymer chains that are compatible with ethylene glycol can also improve the dispersion stability. Branson et al. [92] prepared air-oxidized nanodiamond with an average particle size of ~8 nm in dimethylsulfoxide through bead-milling and ultrasonication, and then mixed the dispersion with glycidol monomer to graft poly(glycidol) polymer brushes on the surfaces of the nanodiamond. They demonstrated that the polymer brush chains were covalently bound to the surfaces of the nanodiamond. Static stable dispersion of the modified nanodiamonds within ethylene glycol fluids with a loading up to 1.9 vol% was achieved. Although detailed dispersion stability at elevated temperatures was not investigated, they reported that the compared with the pure thermal storage fluids the apparent thermal conductivity of the nanofluids could be effectively enhanced.

The polar ethylene glycol fluids also allow for dispersing solar absorbers through electrostatic stabilization. As shown by Figure 4c, Prof. Yu’s team [93] compared the dispersion behavior of multilayer Ti_3_C_2_T_x_ (M-Ti_3_C_2_T_x_) and single-layer Ti_3_C_2_T_x_ (S-Ti_3_C_2_T_x_) MXene within ethylene glycol. They found that the S-Ti_3_C_2_T_x_-EG nanofluids could retain their homogeneous dispersion at room temperature for 30 days and such dispersion stability was much better than that of the M-Ti_3_C_2_T_x_ nanofluids. The enhanced dispersion stability was attributed to more functional groups exposed at the surfaces of the S-Ti_3_C_2_T_x_ and their smaller mass. It was also found that the S-Ti_3_C_2_T_x_ nanofluids had a larger zeta potential than the M-Ti_3_C_2_T_x_ nanofluids under the same pH condition. When the pH value was higher than 7, the zeta potential of the S-Ti_3_C_2_T_x_-EG nanofluids was larger than 30 mV, which is typically considered as a threshold value to achieve long-term electrical stabilization. Although the M-Ti_3_C_2_T_x_ nanofluids had zeta potential values higher than 30 mV, the M-Ti_3_C_2_T_x_ sheets were aggregated meaning that high zeta potential is not the single requirement for achieving stable dispersion. Similarly, Shah et al. [94] reported that introducing surfactants such as sodium dodecyl sulfate (SDS), sodium dodecylbenzene sulfonate (SDBS) and cetyltrimethylammonium bromide (CTAB) could effectively increase the zeta potential of the ethylene glycol nanofluids loaded with RGO particles, but the surface modified RGO still aggregated. Besides zeta potential, it was found that the initial dispersion state, the surface modification process, and the concentration of surfactants and fillers also had significance influence on the dispersion behavior and stability of the nanofluids.

### 4.2. Oil-Based Nanofluids

Compared with ethylene glycol fluids, silicone oil, mineral oil, and other synthetic medium-temperature solar-thermal oils, oil-based nanofluids have a wider range of working temperatures, but they are usually nonpolar. Therefore, hydrogen bonding and electrical stabilization strategies are typically not applicable, and the most frequently adopted approaches are steric stabilization and self-dispersion stabilization in these systems.

**Figure 5:**
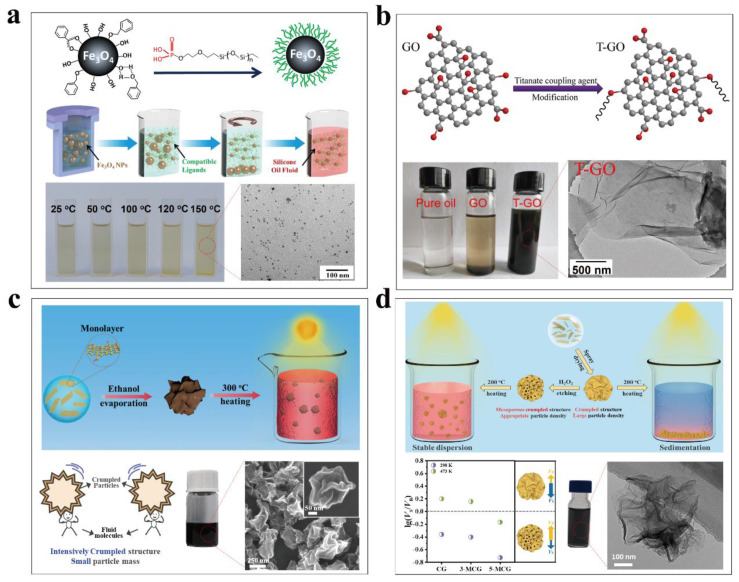
(**a**) Steric stabilization of Fe_3_O_4_ nanoparticles through surface grafting with phosphate-terminated PDMS ligands, which enable their stable uniform dispersion within silicone oil ((**a**) reprinted/adapted with permission from Ref. [95]. 2016, Royal Society of Chemistry). (**b**) Titanate coupling agent modified graphene oxide (T-GO) dispersion within hydraulic oil ((**b**) reprinted/adapted with permission from Ref. [98]. 2017, Elsevier). (**c**) Self-dispersible crumpled RGO particles stably dispersed within silicone oil due to weakened inter-particle van der Waals attraction and gravitational sedimentation ((**c**) reprinted/adapted with permission from Ref. [102]. 2022, Elsevier). (**d**) Mesoporous crumpled graphene particles as self-dispersible solar absorbers within silicone oil ((**d**) reprinted/adapted with permission from Ref. [103]. 2022, Elsevier).

As shown in Figure 5a, Chen et al. [95] modified the surfaces of Fe_3_O_4_ nanoparticles through grafting polydimethylsiloxane (PDMS) chains, which are chemical compatible with the silicone oil thermal storage fluids. The control of the size of the synthesized particles and the robustness of surface binding were critical to achieve stable dispersion. To this end, they utilized benzyl alcohol as the synthetic ligand to control the size distribution of the synthesized Fe_3_O_4_ nanoparticles and further removed the large-sized ones through centrifugation. They synthesized phosphate-terminated PDMS chains as the surface modification agent, which can replace the synthetic ligands and the phosphate head could form robust bidentate binding with the Fe_3_O_4_ nanoparticles. The prepared nanofluids could retain uniform dispersion when they were exposed to direct concentrated solar illumination for harvesting of solar-thermal energy at temperatures higher than 110 °C. Dispersion stability tests indicated that the surface-modified Fe_3_O_4_-silicone oil nanofluids had the tendency to form agglomeration at high particle concentrations and under higher heating temperatures due to increased interparticle colliding probability and desorption of surface modification agents. Similar strategy has also been employed to modify the surface of Fe_3_O_4_@graphene hybrid particles for preparing homogeneously dispersed silicone oil solar-thermal nanofluids [96]. In comparison with Fe_3_O_4_ nanoparticles, the Fe_3_O_4_@graphene hybrid particles had shown improved solar absorptance and could maintain their stable dispersion within silicone oil under a heating temperature up to 150 °C.

Besides long polymer chains, densely grafted short ligands could also provide sufficient steric hindrance to suppress interparticle van der Waals attraction. Gomez-Villarejo et al. [97] adopted an in situ preparation approach and synthesized gold nanoparticles within Dowthermal oil (an eutectic mixture of biphenyl and diphenyl oxide) by using tetraoctylammonium bromide as the surfactant. They found that the surfactant could exchange with diphenyl oxide molecules to stabilize the dispersion of synthesized gold nanoparticles. Although the as-prepared nanofluids were homogeneous, aggregation and sedimentation occurred after heating the nanofluids at 300 °C and those with high concentrations had shown stronger tendency to form agglomerates. To achieve stable dispersion of GO within hydraulic oil, Li et al. [98] modified GO sheets with titanate coupling agents (Figure 5b). The isopropyl triisostearyl titanate ligands could react with hydroxyl groups on the surfaces of GO sheets and the exposed alkyl chains enabled uniform dispersion of modified sheets within hydraulic oil.

In contrast to complex surface modification, transforming planar GO sheets into crumpled RGO particles through an evaporation-induced aerosol compression process offers a facile way to prepare self-dispersible solar-thermal nanofluids [88]. However, the crumpled particles synthesized from the evaporation drying of water-wetted multi-layered GO sheets still agglomerated and precipitated out of thermal storage fluids at elevated temperatures, although they are dispersible at room temperature [88,99,100,101]. In a recent work, Zhang et al. [102] reported that aerosol drying of ethanol-wetted single-layer GO sheets dispersed within ethanol could generate crumpled RGO particles with more intensively deformed surfaces, a smaller particle size, a narrower particle size distribution, and a smaller mass (Figure 5c). The rough wrinkled surfaces prevent direct large-area contact between neighboring particles and thus suppressing van der Waals interparticle attraction. The small size and the small mass favor random Brownian motion of crumpled particles within the thermal storage fluids at elevated temperatures, which could counterbalance the gravitational sedimentation. These features enabled their uniform dispersion within silicone oil when the nanofluids were continuously heated at 200 °C for 14 days. The synthesized particles could be stably dispersed within both polar ethylene glycol and nonpolar oils such as silicone oil and Dowthermal A oil, and the stable operation temperature could reach 300 °C. After revealing such dispersion mechanism, this team further introduced mesopores into the crumpled RGO particles that were synthesized through evaporation drying of water-wetted GO (Figure 5d). They found that the etching of the conventional crumpled particles with hydrogen peroxide solution could lower their apparent density without affecting their original rough surface structure and narrow particle size distribution [103]. By changing the etching duration, the density of the mesoporous crumpled RGO particles could be tailored to match that of the silicone oil so that the Brownian motion could overcome the gravitational sedimentation. The introduced pores can also expose more surface area of the crumpled particles and expand their application field beyond solar-thermal conversion [104].

### 4.3. Ionic Liquid-Based Nanofluids

As a type of liquid salts at room temperature, ionic liquids are composed of organic or inorganic anions such as CF_3_SO_3_^−^, CF_3_CO_2_^−^, and halogen, and organic cations such as imidazolium, pyridinium, pyrazolium, and triazolium [105]. Although ionic liquids show liquid flowing capability and can withstand high operation temperatures, they typically have very low solar absorptance. To improve solar absorptance, various solar absorbers have been incorporated to prepare ionic liquid-based solar-thermal nanofluids with the assistance of steric hindrance stabilization and electrostatic stabilization [106].

**Figure 6:**
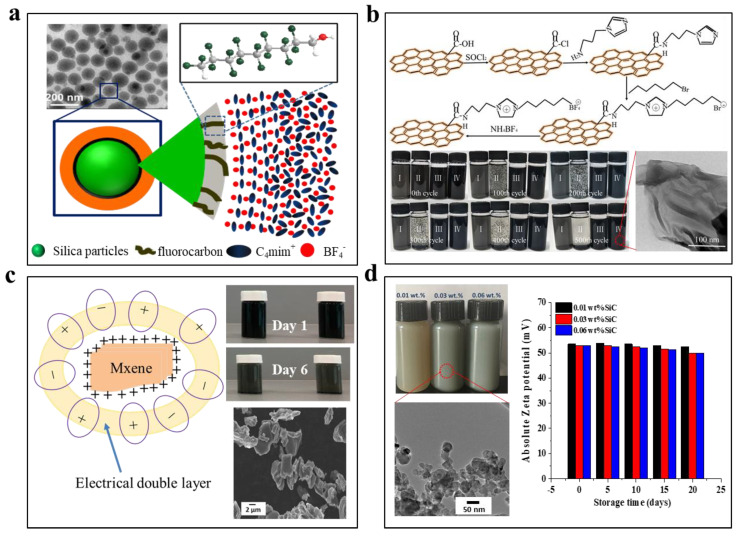
(**a**) Surface modification of SiO_2_ nanoparticles with fluorocarbon brushes to create steric hinderance stabilization and enable their stable dispersion within [C_4_mim]BF_4_ ionic fluids ((**a**) reprinted/adapted with permission from Ref. [108]. 2015, the American Chemical Society). (**b**) Surface modification of graphene by grafting the molecular chains similar to [HMIM]BF_4_ iconic fluids enable stable dispersion of the resultant nanofluids under medium temperatures and cycled heating/cooling conditions ((**b**) reprinted/adapted with permission from Ref. [109]. 2017, Elsevier). (**c**) Homogenous dispersion of MXene (Ti_3_C_2_) within [EMIM][OSO_4_] and diethylene glycol ((**c**) reprinted/adapted with permission from Ref. [111]. 2020, Multidisciplinary Digital Publishing Institute). (**d**) Electrostatically stablized SiC nanoparticles within [HMIM]BF_4_ ionic fluids ((**d**) reprinted/adapted with permission from Ref. [112]. 2017, Elsevier).

Surface modification of solar absorbers to create steric hindrance is the most popular approach to achieve stable dispersion of ionic liquid nanofluids. Dash and Scott [107] reported that trace amount of 1-methylimidazole could serve as the stabilizer to homogenize the dispersion of as-synthesized gold nanoparticles when they reduced HAuCl_4_ with NaBH_4_ within imidazolium-based ionic liquids. Moreover, they found that the binding of 1-methylimidazole ligands helped improving the monodispersity of synthesized particles and such dispersion strategy was also applicable to bimetallic nanoparticles. As shown in Figure 6a, Gao et al. [108] covalently grafted 9-carbon chain fluorocarbon onto the surfaces of SiO_2_ nanoparticles and achieved stable colloidal dispersion within 1-butyl-3-methylimidazolium tetrafluoroborate [C_4_mim][BF_4_]. They further analyzed that the grafted fluorocarbon layer had a thickness of ~1 nm, which could not provide sufficient steric hindrance to screen the interparticle attraction. It was revealed that it was the solvation layer surrounding the surfaces of the modified particles, which had a thickness of ~5 nm, that provided the sufficient steric stabilization.

To disperse graphene within ionic liquids, Zhang’s group [109] firstly prepared carboxyl-terminated graphene (GE-COOH) and then grafted molecular chains that were similar to the 1-hexyl-3-methylimidazolium tetrafluoroborate ([HMIM]BF_4_) by using the same synthetic reagents for synthesis of ionic liquids (Figure 6b). The modified graphene could retain their uniform dispersion when the nanofluids were subject to both continuous heating at 180 °C for one month and repeated heating/cooling from 30 °C to 180 °C for 500 cycles. By contrast, without surface grafting the GE-COOH demonstrated poor dispersion stability and precipitated out of the ionic liquids after the heating tests. The stable dispersion was attributed to the compatibility of grafted chains with ionic liquids and the high zeta potential of the modified graphene. They also reported that under the same concentration graphene had shown superior enhancement of the key thermophysical properties of the ionic liquids including thermal conductivity, solar absorptance, and photothermal conversion efficiency than graphite nanoparticles and single-wall CNTs [110].

In parallel with surface modification with stabilizing ligands, introducing charges to the surfaces of solar absorbers can induce the formation of electrical double layer to stabilize their dispersion within ionic liquids. For example, Bakthavatchalam et al. [111] prepared multi-layer MXene through a chemical etching route and dispersed them within the mixture of diethylene glycol and 1-ethyl-3-methyl imidazolium octyl sulfate ([Emim][OSO_4_]) ionic liquid. The resultant nanofluids had shown stable uniform dispersion during the first 4 days. In this case, the charged MXene surfaces attract [Emim] cations and [OSO_4_] anions, which reorganized into an electrical double layer and prevented aggregation (Figure 6c). However, slight aggregation and sedimentation were observed at days 5, 6, and 7. In another work, Chen et al. [112] reported that SiC nanoparticles could be uniformly dispersed within [HMIM]BF_4_ ionic liquids without observing obvious settlement for 20 days (Figure 6d). The absolute values of zeta potential of the prepared nanofluids were measured to be higher than 50 mV, which supported the electrostatic stabilization mechanism. It should be noted that previous theoretical and experimental studies indicated that the high charge density of ionic liquids strongly screens the electrostatic repulsion between neighboring particles and the thickness of electrical double layer is less than 1 nm, which is too thin to provide sufficient electrostatic stabilization [113,114]. Therefore, the dispersion stability mechanism of the electrostatically stabilized ionic nanofluids needs more detailed investigation. Moreover, all these reported dispersion tests were carried out at room temperature, and their dispersion behaviors at elevated temperatures need to be further investigated.

### 4.4. Molten Salt-Based Nanofluids

As a cousin of organic ionic liquids, inorganic molten salts not only possess high charge density but also require higher heating temperatures to keep them in the liquid state, which together pose even grand challenges in achieving stable dispersion of functional nanofillers. Within melted molten salts, the resultant Debye screening length is only ~0.1 nm, which limits the application of classical electrostatic stabilization strategy. The high operation temperature restricts the employment of steric stabilization through surface modification organic ligands that might degrade over time.

**Figure 7:**
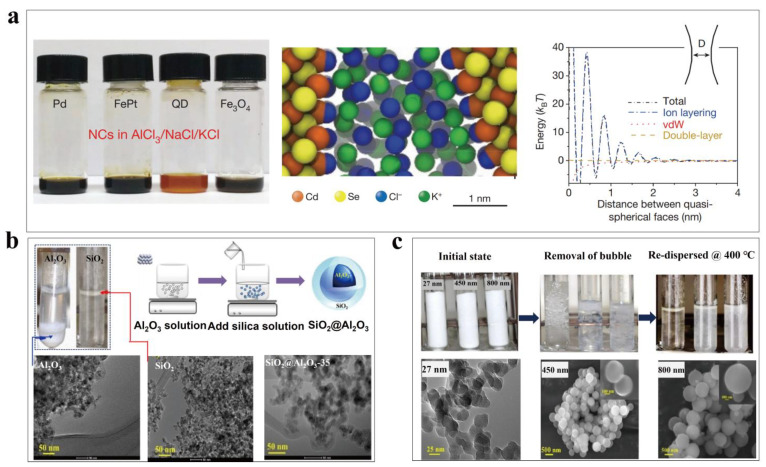
(**a**) Homogeneous dispersion of colloidal nanoparticles within molten salts due to chemical affinity between the nanoparticle surfaces and the molten salt ions ((**a**) reprinted/adapted with permission from Ref. [115]. 2017, Springer Nature). (**b**) Core-shell SiO_2_@Al_2_O_3_ nanoparticles dispersed within melted solar salts due to reduced sedimentation tendency through matching the density of particles and molten salts. ((**b**) reprinted/adapted with permission from Ref. [119]. 2019, Elsevier). (**c**) Dependence of nanofluid dispersion stability on the size of SiO_2_ particles ((**c**) reprinted/adapted with permission from Ref. [120]. 2022, Elsevier).

In recent years, Talapin’s [115,116] group reported a strategy to disperse functional nanofillers including metals (Pt, Pd), semiconductors (CdSe/CdZnS quantum dots), rare-earth compounds (NaYF_4_:YbEr/CaF_2_) and magnetic particles (Fe_3_O_4_) in molten salts. The chemical affinity between the particle surfaces and the ions in the molten salts was identified as the key enabler to achieve stable uniform dispersion. For example, Pt and Pd nanoparticles could be homogeneously dispersed within the mixed AlCl_3_/NaCl/KCl molten salts when there was excessive AlCl_3_ because the electron-rich transitional metals (Pt, Pd) could form chemical bonds with AlCl_3_ (Figure 7a)_._ Without excessive AlCl_3_ in the molten salts, severe agglomeration of Pt particles was observed. Similarly, CdSe nanoparticles could be dispersed within the Lewis-basic halide salts such as AlCl_3_/NaCl/KCl and LiCl/LiI/KI, and the pseudohalide salts such as NaSCN/KSCN as well because the halide and SCN^−^ ions could efficiently bind with Cd^2+^ at the external surfaces of CdSe particles. Molecular dynamic simulation indicated that Cl^−^ ions formed a dense epitaxial layer and the co-ions were organized into a structured ion layer. The repulsive interaction between the ion layer is much stronger than both the van der Waals and double-layer electrostatic interaction, proving its dominant contribution to stable dispersion. Although this pioneering work provided a feasible approach to disperse various functional nanoparticles within molten salts, it is worth mentioning that the demonstrated salts typically have a relatively low melting temperature and the long-term stability of added particles at elevated temperatures has not been evaluated.

In solar-thermal storage research directions, intensive efforts were mainly devoted to incorporating SiO_2_, Al_2_O_3_, MgO, and other nanoparticles into commercial solar-thermal molten salts that have been employed for thermal storage at medium-to-high temperatures [117,118]. For example, Nithiyanantham et al. [119] found that while pure Al_2_O_3_ and SiO_2_ particles precipitated or floated within solar salts (NaNO_3_-KNO_3_) after heating the nanofluids at 400 °C for 24 h (Figure 7b), the core-shell SiO_2_@Al_2_O_3_ could create steric hindrance and obtain an intermediate density matching that of the molten salts thereby reducing agglomeration and sedimentation tendency. They utilized Al_2_O_3_ particles with a diameter of 12 nm as the core, and tailored the SiO_2_ shell thickness through changing the concentration of silica precursor. It was found that the chain-like slightly aggregated SiO_2_@Al_2_O_3_ could effectively enhance thermal conductivity of the nanofluids, but in the meanwhile caused higher viscosity. In a subsequent work [120], this team reported that the dispersion of SiO_2_ particles within melted solar salts also depended on the particle size (27 nm, 450 nm, 800 nm) and larger-sized SiO_2_ particles (800 nm) have demonstrated better dispersion (Figure 7c). They found that the dispersion stability and enhancement of thermophysical properties of the nanofluids (specific heat capacity, thermal conductivity) have opposite dependence on the size of SiO_2_ particles.

It is worth pointing out that so far most of the reported works mainly focused on improving heat capacity and thermal conductivity of the molten salts, and the prepared nanofluids typically have low solar absorptance. Additionally, the dispersion behavior and governing mechanisms of various particles within the molten salts over a long period of operation time are awaiting further exploration. It remains a grand challenge to prepare stably dispersed molten salt nanofluids with boosted thermophysical properties for direct solar-thermal energy harvesting at medium-to-high temperatures.

## 5. Summary and Outlook

In summary, dispersion stability has been viewed as one of the long-lasting intractable issues limiting the development and practical application of nanofluid technologies. Recent years have witnessed substantial progress in understanding the dispersion behavior and developing effective strategies to prepare medium-temperature solar-thermal nanofluids homogeneously dispersed with a variety of functional nanofillers. Key thermophysical properties including solar absorptance, thermal conductivity, and specific heat capacity have shown significant enhancement. It is anticipated that these dispersed nanofluids hold the promise for expanding the usage of DASCs from low-temperature into medium-temperature solar-thermal systems with improved energy harvesting efficiency. From a broader perspective, the aggregation-resistant solar-thermal nanofluids will also enable other wide-spread thermal-related applications such as heat transfer in renewable energy systems, and thermal management of electronic devices [121].

Although the benefits for improving volumetric direct solar-thermal conversion have been widely demonstrated by the uniformly dispersed nanofluids, the influence of added solar absorbers on other important thermophysical properties such as viscosity and effective thermal conductivity has not been fully revealed [122]. Typically, the viscosity of nanofluids increases with added fillers and is affected by their loading, size, shape, dispersion state, and surface chemistry [123]. In comparison with bare fillers, the surface-modified particles could generally minimize the increasing of viscosity because these surface-capping agents improve their chemical compatibility with the thermal storage fluids. Another fact is that the viscosity of the nanofluids quickly drops with increasing application temperature. Such temperature dependence implies that the medium-temperature solar-thermal nanofluids, which have a low loading of dispersed solar absorbers, should have largely the same viscosity as the neat thermal storage fluids at elevated temperatures, and the effective viscosity can be roughly estimated by the classical Einstein model. Regarding the effective thermal conductivity of the nanofluids, most frequently the added particles have a higher thermal conductivity than the thermal storage fluids and thus can help improve the thermal conductivity and heat transfer performance. For uniform dispersion at low loading levels, the effective thermal conductivity can be estimated by the effective medium theory or many other classical models [124], which generally predict that the effective thermal conductivity is positively dependent on the volumetric fraction of the added fillers. It was often observed that increasing temperature is favorable for intensifying interparticle collision and local convection heat transfer, and thus is helpful for improving the effective heat conductivity [125]. In general, self-dispersible nanofluids should possess better heat transfer performance than the surface-modified nanofluids as the surface-capping agents typically have a low thermal conductivity and limit interparticle collision heat transfer. It should be noted that measurements of both the viscosity and the thermal conductivity of solar-thermal nanofluids currently most often were only performed at low temperatures. It is highly desired that we can take advantage of recent progresses in preparing stably dispersed medium-temperature solar-thermal nanofluids to carry out systematic characterization of the thermophysical properties at elevated temperatures and reveal the influence of added fillers on the properties of the nanofluids.

To fulfill the potential of medium-temperature nanofluid technologies, continuous research efforts from different disciplines are needed to comprehensively reveal the long-term dispersion behavior of nanofluids at elevated temperatures, establish the relationship between dispersion state and enhancement of thermophysical properties, and systematically evaluate the impact of these nanofluids on the improved performances of solar-thermal systems. To this end, different dispersion characterization techniques such as dynamic light scattering [126] and in situ transmission electronic microscopy [127,128] operating at high temperatures should be developed to facilitate the monitoring the dynamic dispersion and evolution of nanofillers within the medium-temperature nanofluids at different dimensional scales. Computational modeling tools such as molecular dynamic simulations have demonstrated unique advantages in evaluating particle-fluid and interparticle interaction at molecular and atomic levels [115]. These techniques should be combined with experimental investigation to fully elucidate the dispersion mechanisms. Another important future research direction would be developing advanced setups or tools to experimentally characterize the thermophysical properties of solar-thermal nanofluids at medium-to-high temperatures. To enable practical applications in current solar-thermal industries, on-going research efforts should be focused on the development of self-dispersible synthetic oil and molten salt-based nanofluids loaded with low concentrations of solar absorbers. Manufacturability of solar absorbers at large scale, low cost through one-step process should also be considered [129].

## 6. Conclusions

This work overviews recent progress in preparing medium-temperature ethylene glycol, oil, ionic liquid, and molten salt-based solar-thermal nanofluids that are uniformly dispersed with various types of solar absorber. Four categories of stabilization strategies including hydrogen bonding stabilization, electrostatic stabilization, steric stabilization, and self-dispersive stabilization have shown the capability to improve the dispersion of solar absorbers within the thermal storage fluids. By providing representative examples, the applicable dispersion strategies for different thermal storage fluidic systems have been identified. Among them, hydrogen bonding stabilization has appeared to be a facile effective way to prepare uniformly dispersed ethylene glycol-based medium-temperature nanofluids. Although the surfaces of solar absorbers could be modified with organic ligands or polymeric chains to improve their dispersion within medium-temperature oils, self-dispersion strategy has exhibited unique advantages in simple preparation processes and long-term dispersion stability. Electrostatic stabilization and self-dispersion stabilization are promising for preparing uniformly dispersed ionic liquid and molten salt-based solar-thermal nanofluids. In comparison, recently emerged self-dispersible nanofluids have demonstrated superior dispersion stability and possess unique promises to improve practical solar-thermal energy harvesting performances without sacrificing the original heat capacity and low viscosity features of the thermal storage fluids. It thus merits further development of diverse self-dispersion strategies, controllable fabrication of self-dispersible solar absorbers, and carrying out systematic investigation of their thermophysical properties and full evaluation of their direct absorption solar-thermal harvesting performances. It is hoped that the reviewed research progress in medium-temperature nanofluids and these identified research needs provide many exciting opportunities for cross-discipline collaboration to advance further development of nanofluid technologies.

## Data Availability

Not applicable.
